# The use of repeated blood pressure measures for cardiovascular risk prediction: a comparison of statistical models in the ARIC study

**DOI:** 10.1002/sim.7144

**Published:** 2016-10-11

**Authors:** Michael J. Sweeting, Jessica K. Barrett, Simon G. Thompson, Angela M. Wood

**Affiliations:** ^1^ Department of Public Health and Primary Care, School of Clinical Medicine University of Cambridge Cambridge U.K.

**Keywords:** repeat measures, cardiovascular risk prediction, joint models, C‐index, regression calibration

## Abstract

Many prediction models have been developed for the risk assessment and the prevention of cardiovascular disease in primary care. Recent efforts have focused on improving the accuracy of these prediction models by adding novel biomarkers to a common set of baseline risk predictors. Few have considered incorporating repeated measures of the common risk predictors. Through application to the Atherosclerosis Risk in Communities study and simulations, we compare models that use simple summary measures of the repeat information on systolic blood pressure, such as (i) baseline only; (ii) last observation carried forward; and (iii) cumulative mean, against more complex methods that model the repeat information using (iv) ordinary regression calibration; (v) risk‐set regression calibration; and (vi) joint longitudinal and survival models. In comparison with the baseline‐only model, we observed modest improvements in discrimination and calibration using the cumulative mean of systolic blood pressure, but little further improvement from any of the complex methods. © 2016 The Authors. *Statistics in Medicine* Published by John Wiley & Sons Ltd.

## 1 Introduction

Primary prevention of cardiovascular disease (CVD) in individuals is centred on the use of risk prediction equations to target preventive interventions, such as lifestyle and pharmacological treatments, to people who should benefit most from them. These algorithms estimate risk of CVD events from prediction models that incorporate information on several risk factors, such as age, sex, smoking habits, history of diabetes mellitus, and levels of systolic blood pressure (SBP) and serum lipids. Recently, most research has focused on improving the accuracy of these prediction models by including novel biomarkers 1 or a broader set of predictors using available information in electronic health records (e.g. QRISK^2^).

However, most CVD risk algorithms have been derived using risk predictors measured at a single time point. If the risk factor is volatile (i.e. within‐person variability is high) or measured with error (e.g. SBP), then using a single measurement will lead to imprecise risk predictions. Furthermore, incorporating knowledge regarding the rate of change in a biomarker over time may also improve CVD risk prediction. Much of the previous research into the benefit of including repeat measurements in CVD risk prediction has focused on a single repeat measurement 3, 4.

Developing a CVD risk prediction algorithm generally involves fitting a time‐to‐event survival model to a prospectively collected cohort of, initially, disease‐free individuals. To incorporate a risk factor, or biomarker, that varies over time requires a more complex statistical model or simplifying assumptions regarding how the measured biomarker, or its underlying level, changes over follow‐up and its effect on the hazard of developing the disease of interest. One common approach is to use a survival model with a time‐dependent covariate, but for biomarkers that are measured intermittently, the hazard at any time point is calculated based on an individual's last observation carried forward (LOCF), which could be an inaccurate assessment of their current biomarker value. Furthermore, standard survival models do not account for measurement error in covariates. Recently, there has been much interest in the use of joint models for longitudinal and survival data to model the underlying, error‐free, biomarker trajectory and disease processes simultaneously and hence overcome the limitations of standard time‐dependent survival models as well as the issue of informative drop‐out due to the event 5, 6, 7. Joint models have been demonstrated to provide unbiased estimates of hazard ratios in contrast with survival models that use time‐dependent covariates 5, 6. In terms of survival predictions, Yang *et al.* have recently found increased predictive performance for a variety of joint models compared with baseline‐only and time‐dependent Cox models 8. However, the comparison of joint models with other simpler models that utilise repeat measures is under‐researched.

In this paper, we illustrate the use of a number of prediction models that attempt to incorporate repeat measures of a single biomarker. These range from the ‘baseline‐carried forward’ method using a survival model with the biomarker as a time‐dependent covariate, to a simple survival model that incorporates a function of the previously observed biomarker measurements, and to more complex methods that attempt to model the biomarker process. We illustrate these methods using data on repeat SBP measurements from the Atherosclerosis Risk in Communities (ARIC) study. We obtain dynamic 10‐year CVD risk predictions and assess these using measures of predictive discrimination and accuracy. The danger in obtaining over‐optimistic risk predictions is highlighted and addressed through the use of a (internal) validation cohort.

Findings from these investigations are then further explored through the use of simulation studies. Finally, we conclude with a discussion regarding any added benefit of using repeat SBP measurements in the context of CVD risk prediction, how results may apply more generally to risk prediction and the limitations of these investigations.

## 2 Prediction models

We consider a study that recruits *n* patients. Each patient *i* is followed up from baseline until $T_{i}^{*}=\min(T_{i},C_{i})$, where *T*_*i*_ is the event time of interest and *C*_*i*_ is a censoring time. In addition to a set of baseline predictors *Z*_*i*_, which we will assume are known without error (e.g. age, sex and history of diabetes), let *X*_*i*_(*t*) denote a single time‐varying predictor, for example, a biomarker such as SBP, measured at time *t*. Interest lies in the *L*‐year risk prediction of developing the event in the time horizon [*t*_*p*_,*t*_*p*_ + *L*), given survival up to *t*_*p*_. To do this, consider the following survival model with a time‐varying predictor:

(1) $$h_{i}(t)=h_{0}(t)\exp(H_{t}{\left(X_{i}\right)}^{T}\alpha +Z_{i}^{T}\gamma ),$$

where $H_{t}(X_{i})$ is a function, or possibly vector of functions, of the history of the biomarker process up to time *t*. The baseline hazard *h*_0_(*t*) can either take a parametric form or be left unspecified. The aim of this work is to fit the survival model once using all follow‐up data in the cohort and subsequently to make risk predictions at any given time *t*_*p*_ for individuals still at risk (i.e. $T_{i}^{*}>t_{p}$). This is in contrast to the landmarking approach 9, which explicitly separates the ‘past’ biomarker measurements from the ‘future’ survival follow‐up at the prediction time. We compare the two approaches in more detail in Section 6.

Some issues arise that often preclude us from fitting Equation ((1)) to our data. Firstly, the biomarker is commonly an imprecise measurement of the underlying risk factor of interest, such that we may observe *X*(*t*) = *X* ^∗^(*t*) + *ε*(*t*), where *X* ^∗^(*t*) is the true error‐free biomarker and *ε*(*t*) represents within‐person variability. Secondly, the biomarker is usually only measured at certain examination times, $t_{i0},\ldots ,t_{im_{i}}$, where *m*_*i*_ is the number of post‐baseline examinations for patient *i*. Therefore, in reality, the data obtained consist of $\left\{Z_{i},X_{i}(t_{i0}),\ldots ,X_{i}(t_{im_{i}})\right\}$, and hence, the full underlying trajectory is unknown.

In practice, models that simplify Equation ((1)) are usually fitted. We now consider a series of such models of increasing complexity. In what follows, we shall ignore the issue of competing risks when making risk predictions; however, such methodology could easily be incorporated if required.

### 2.1 Model I: baseline carried forward

The simplest model to be considered utilises only observed baseline information on all predictors and assumes time‐homogeneous effects. This is the model that has been used to develop the cardiovascular risk scores most commonly implemented in current clinical practice when no repeat measures are available 2, 10. Because the baseline values are assumed to act on the hazard throughout follow‐up, we will refer to this model as a baseline carried forward (BCF) model. This model uses the ‘uncorrected’ observed measurements, and hence, estimated regression coefficients are likely to be diluted because of measurement error (e.g. laboratory error) 9. In addition, the BCF model does not account for any ‘ageing’ covariates (i.e. those that are time dependent in nature) 9. In Equation ((1)), $H_{t}(X_{i})$ is replaced with $H_{t}^{\left(BCF\right)}(X_{i})$ where

$$H_{t}^{\left(BCF\right)}(X_{i})=X_{i}(0)\;\text{for all}\;t$$

and *X*_*i*_(0) denotes the observed baseline value of the predictor *X* for patient *i*.

### 2.2 Model II: last observation carried forward

A relatively common method and one that is easily implemented in standard statistical software is to use an LOCF approach, whereby the observed time‐varying biomarker is used as a time‐dependent covariate in the survival model but is assumed to remain constant in the hazard function between examination times. This model is likely to provide more accurate estimates of the regression coefficients because the hazard is updated at each examination using more recent (and hence relevant) covariate information. However, the model still uses the observed values as if they were measured without error. In Equation ((1)), $H_{t}(X_{i})$ is now replaced with $H_{t}^{\left(LOCF\right)}(X_{i})$ where

$$H_{t}^{\left(LOCF\right)}(X_{i})=X_{i}\left(t_{ik}\right),$$

and where $t_{ik}=\max\left\{t_{ij}|t_{ij}⩽t\right\}$ for *j* = 0,…,*m*_*i*_.

### 2.3 Model III: cumulative average

One possible extension of the LOCF method is to allow the biomarker predictor to be a function of the history of the observed biomarker measurements. This is a simple approach that can start to deal with measurement error and long‐term within‐person variation in the biomarker. An average of the previous measurements is one possible predictor. The average represents an unbiased estimate of the true mean given no underlying trend, bias or heteroskedasticity in the process. The time‐varying predictor is then defined by a cumulative average (CA) as follows:

$$H_{t}^{\left(CA\right)}(X_{i})=\frac{\overset{m_{i}}{\underset{j=0}{\sum }}I(t_{ij}<t)X_{i}(t_{ij})}{\overset{m_{i}}{\underset{j=0}{\sum }}I(t_{ij}<t)},$$

where *I*(·) is the indicator function. This predictor is then used in place of $H_{t}(X_{i})$ in Equation ((1)).

Further extensions to this model could be envisaged whereby measurements taken further back in time are discounted (or down‐weighted) to create a weighted CA predictor, for example, an exponentially weighted moving average.

### 2.4 Model IV: ordinary regression calibration

#### 2.4.1 Stage 1

A two‐stage or regression calibration approach attempts to model the time‐varying biomarker process using a linear mixed model and then, in a second stage, plugging the predicted response into the survival model 11, 12. More formally, consider the mixed‐effects model:

(2) $$\begin{array}{cc} X_{i}(t) & =X_{i}^{*}(t)+\varepsilon_{i}(t)=P_{i}{\left(t\right)}^{T}\beta +Q_{i}{\left(t\right)}^{T}b_{i}+\varepsilon_{i}(t)\\ b_{i} & ∼N(0,\Sigma ),\;\varepsilon_{i}(t)∼N\left(0,\sigma_{\varepsilon }^{2}\right), \end{array}$$

where *P*_*i*_(*t*) and *Q*_*i*_(*t*) are vectors of explanatory variables for the fixed and random effects, respectively, and may include any of the baseline predictors *Z*_*i*_. For example, the commonly used random‐intercept, random‐slope model is specified with *P*_*i*_(*t*) = (1,*t*,*Z*_*i*_)^*T*^ and *Q*_*i*_(*t*) = (1,*t*)^*T*^, such that

(3) $$X_{i}(t)=\beta_{0}+\beta_{1}t+b_{0i}+b_{1i}t+Z_{i}^{T}\beta_{2}+\varepsilon_{i}(t).$$

From the mixed‐effects model, we can obtain the maximum likelihood estimates of the fixed‐effect parameters and the best linear unbiased predictors of the random effects, ${\hat{b}}_{i}$. An ordinary regression calibration (ORC) approach does this by fitting a single mixed‐effects model using all individuals and data 12, from which we can then obtain the predicted values of *X* ^∗^(*t*):

$$X_{i}^{\left(ORC\right)}(t)=E\left(X_{i}^{*}(t)|X_{i},P_{i}(t),Q_{i}(t),\hat{\beta },\hat{\Sigma }\right),$$

where $X_{i}=\left(X_{i}(t_{i0}),\ldots ,X_{i}(t_{im_{i}})\right)$ is the complete vector of measurements for patient *i*.

#### 2.4.2 Stage 2

In the second stage, any function of the estimated fixed and random effects, $\psi \left(t;\hat{\beta },\hat{\Sigma },{\hat{b}}_{i}\right)$, can be used as a time‐dependent predictor in the survival model, replacing $H_{t}(X_{i})$ in Equation ((1)). This function could, for example, be the predicted values $X_{i}^{\left(ORC\right)}(t)$, or indeed, the random effects themselves could be used in which case there would be as many association parameters as there are random effects. This function, or vector of functions ***ψ***, is then plugged into the survival model as follows:

(4) $$h_{i}(t)=h_{0}(t)\exp\left(\psi {\left(t;\hat{\beta },\hat{\Sigma },{\hat{b}}_{i}\right)}^{T}\;\alpha +Z_{i}^{T}\gamma \right).$$

A Cox proportional hazards survival model only requires the evaluation of the time‐varying predictor at the unique event times because the partial likelihood is a product of terms over the event times. Hence, for each individual, follow‐up should be partitioned at the unique event times and the time‐varying predictor evaluated at each. However, for parametric survival models, an approximation to the likelihood can be made by partitioning follow‐up into a number of small time intervals, and assuming the time‐dependent predictor remains constant within each of these intervals 11.

### 2.5 Model V: risk‐set regression calibration

An alternative approach, called risk‐set regression calibration (RRC) 13, fits multiple mixed‐effects models, one for each of the unique event times in the dataset. For an event time, *T*_*k*_, only individuals still at risk are included in the mixed model $\left\{i:T_{i}^{*}>T_{k}\right\}$, and only the biomarker measurements of these individuals up to that event time are used $X_{i(T_{k})}=\left\{X_{i}(t_{ij}):t_{ij}<T_{k}\right\}$. Then the predicted value of the biomarker for individuals still at risk at this event time can be calculated to give the following:

$$X_{i}^{\left(RRC\right)}(T_{k})=E\left(X_{i}^{*}(T_{k})|\;X_{i(T_{k})},Z_{i},\hat{\beta },\hat{\Sigma },T_{i}^{*}>T_{k}\right).$$

A similar derivation can be used to obtain the empirical Bayes estimates of the random effects, which now change at each of the event times, ${\hat{b}}_{i}(T_{k})$. This contrasts with the ORC approach where the estimated random effects for an individual stay constant over follow‐up. Aside from this difference, stage 2 of the RRC approach then proceeds exactly as described in the ORC approach.

The RRC approach has been shown to yield estimators for the hazard ratios in Equation ((1)) that reduce but do not eliminate bias relative to more naive methods 14, 15. One reason for this is that the empirical Bayes estimator of the predicted biomarker in individuals still under follow‐up after a period of time may not be normally distributed 6.

### 2.6 Model VI: a joint longitudinal and survival model

A drawback of a two‐stage approach is that the uncertainty in the estimated fixed and random effects is not carried forward to the survival model, resulting in estimates that are too precise 11. Event‐dependent drop‐out may also cause bias in the ORC two‐stage approach 12, 16, 17, while the RRC‐predicted values may be more variable, especially towards the end of follow‐up when fewer individuals are left in the risk‐set.

A joint model attempts to model both the longitudinal trajectory and the survival data simultaneously in a one‐stage approach using a shared random‐effects model 5, 7. The longitudinal sub‐model and survival sub‐model are specified as in Equations ((2)) and ((4)), and the likelihood function considers the joint density of both outcomes. To proceed with maximising the likelihood, it is assumed that the two outcomes are conditionally independent given the random effects.

## 3 Dynamic risk prediction

Each of the six models described in Section 2 can be used to predict the risk of an event occurring within a prediction window for a new individual with a history of biomarker measurements. These risk predictions may be updated when further measurements are taken, leading to dynamic risk predictions that change over time as more data accumulate.

To avoid numerical integration of a time‐dependent hazard function, we investigate risk predictions based on linear predictors that are time constant over the horizon of the risk prediction. For models I–III, this is achieved by making a prediction based only on biomarker measurements taken before the start of the prediction window. For models IV–VI, the linear predictor of the survival sub‐model is time constant when the association structure uses the random effects directly. Predictions from a RRC model use the latest time‐updated random effects prior to the beginning of the prediction window. Hence, the *L*‐year risk prediction within the prediction window [*t*_*p*_,*t*_*p*_ + *L*), given survival up to *t*_*p*_, is calculated as follows:

$$\begin{array}{cc} P_{i}^{L}(t_{p}) & =P\left(T_{i}⩽t_{p}+L|T_{i}>t_{p},X_{i(t_{p})},Z_{i}\right)\\ =1-{\left(\frac{{Ŝ}_{0}(t_{p}+L)}{{Ŝ}_{0}(t_{p})}\right)}^{\exp\left(\psi_{i}{\left(t_{p}\right)}^{T}\;\hat{\alpha }+Z_{i}^{T}\;\hat{\gamma }\right)}, \end{array}$$

where *ψ*_*i*_(*t*_*p*_) is substituted with the appropriate prediction depending on which model is chosen and is equal to $H_{t_{p}}^{\left(M\right)}(X_{i})$ for *M* = {*B* *C* *F*,*L* *O* *C* *F*,*C* *A*} (models I–III) and $\left\{{\hat{b}}_{0i}(t_{p}),{\hat{b}}_{1i}(t_{p})\right\}$ for the ORC, RRC and joint models (models IV–VI), assuming a random‐intercept random‐slope model as in Equation ((3)).

To compare the predictive performance of models I–VI, we use both discrimination and calibration measures of predictive accuracy. Discrimination measures assess how well the model discriminates between individuals, while calibration measures assess the accuracy of individual risk predictions.

### 3.1 Discrimination

The C‐index 18 is commonly used as the predictive metric to assess discrimination. For dynamic risk prediction, a dynamic C‐index can be calculated, which considers concordance within the prediction window of interest and is calculated using only those individuals still at risk at the start of the prediction window 19. Let D be the set of unique event times, *R*(*t*_*i*_) the risk‐set at event time *t*_*i*_ and *Y*(*t*_*i*_) the size of the risk‐set at *t*_*i*_. Then the C‐index associated with a *L*‐year risk prediction from time *t*_*p*_ is defined as follows:

$$C^{L}(t_{p})=\frac{\underset{i\in D;t_{p}⩽t_{i}⩽t_{p}+L}{\sum }\left(\#\left\{j\in R(t_{i});P_{j}^{L}(t_{p})<P_{i}^{L}(t_{p})\right\}+0.5\cdot \#\left\{j\in R(t_{i}),j\neq i;P_{j}^{L}(t_{p})=P_{i}^{L}(t_{p})\right\}\right)}{\underset{i\in D;t_{p}⩽t_{i}⩽t_{p}+L}{\sum }\left(Y(t_{i})-1\right)}.$$

The C‐index compares the concordance of the risk predictions from all possible pairs of ‘useable’ individuals (*i.e.* pairs in which at least one individual has an event within the prediction window of interest) against the ordering of the observed event times. A C‐index of 0.5 indicates no discriminative ability, beyond that of chance, while a value of 1 indicates perfect concordance between the prognostic model and the empirical event times.

### 3.2 Calibration

Calibration is assessed using Brier scores and calibration plots, also calculated dynamically using only those at risk at the start of the prediction window. At the end of the prediction window, those still at risk are censored 20.

The Brier score measures the mean‐squared prediction error, weighted to account for censored observations 21, where lower values of the Brier score represent better calibrated predictions. Let *δ*_*i*_ denote the event indicator *δ*_*i*_ = *I*(*T*_*i*_⩽*C*_*i*_), and let ${Ĝ}_{R(t_{p})}(t)$ be the Kaplan–Meier estimate of the probability of censorship survival for those at risk at *t*_*p*_. Then the Brier score is defined as follows:

$$BS^{L}(t_{p})=\frac{1}{\#\left\{j\in R(t_{p})\right\}}\underset{i\in R(t_{p})}{\sum }{\left(P_{i}^{L}(t_{p})-I(T_{i}⩽t_{p}+L)\right)}^{2}\omega_{i}(t_{p},L)$$

where

$$\omega_{i}(t_{p},L)=\frac{I(T_{i}⩽(t_{p}+L))\delta_{i}}{{Ĝ}_{R(t_{p})}(T_{i})}+\frac{I(T_{i}>(t_{p}+L))}{{Ĝ}_{R(t_{p})}(t_{p}+L)}.$$

Calibration plots display the observed risk of an event against the mean‐predicted risk within (for example) deciles of the predicted risk. The observed risk of an event is estimated using a Kaplan–Meier estimate of the survivor function, stratified by decile group. The better calibrated a model, the closer the calibration curve will lie to the diagonal.

## 4 Applying the models to the Atherosclerosis Risk in Communities study to assess cardiovascular disease risk prediction

The ARIC study is an ongoing prospective cohort study with approximately 13 000 individuals who were free of prevalent CVD. Individuals aged 45–64 years were recruited into the study between 1987 and 1989. Our dataset included follow‐up for CVD events until December 2011. Paynter *et al.* have previously investigated the use of regression calibration in the ARIC study using data from a baseline visit and a single repeat visit 3.

The ARIC study collected extensive baseline information (including medical, social and demographic), and participants had clinical biomarkers, such as blood pressure and cholesterol re‐measured at three further follow‐up visits through 1996 to 1998, taken approximately 3 years apart. There were 2340 CVD events over a median follow‐up of 22.3 years. We consider a standard set of CVD risk factors in order to develop our risk prediction models: age, sex, smoking status, history of diabetes, SBP, total cholesterol and high‐density lipoprotein (HDL)‐cholesterol. Any participants missing baseline measurements for these risk factors were excluded leaving *n* = 13 153 individuals for analysis. In addition, we included repeat measures of SBP taken at each of the follow‐up examinations in order to compare models that utilised these repeat measurements. Individuals who died from non‐CVD causes were censored at their time of death.

### 4.1 Models

In order to compare models I–VI described in Section 2, we consider models with a flexible parametric baseline hazard function in which the log cumulative hazard is modelled using restricted cubic splines 22. These models can be fit in Stata using the stpm2 function 23 and the stjm function for a joint model with a flexible parametric baseline hazard 24. All models used restricted cubic splines with two knots placed at the tertiles of the distribution of uncensored log event times.

In the two‐stage and joint modelling approaches, models IV–VI, a random‐intercept random‐slope model was considered for the repeated SBP values over time, adjusted for baseline values of the remaining standard CVD risk factors. More complex models were investigated but rejected because of lack of parsimony in a dataset with only four repeat measurements. All three models used both the random intercept and slope as direct association parameters in the survival sub‐model, thus allowing risk predictions to be made based on a time‐constant linear predictor (Section 3).

Furthermore, for computational convenience, we fitted the RRC mixed models only for three subsets of the data: (i) for all individuals with at least one repeat measurement using SBP measurements at baseline and first repeat; (ii) for all individuals with at least two repeat measurements using SBP measurements up to and including the second repeat; and (iii) for all individuals with all three repeat measurements using all SBP measurements. Predictions could not be made from baseline for the RRC model, because at least two repeat SBP measurements are required to fit the mixed model. This is a simplification of the true RRC approach in which a separate mixed model is estimated at each event time. Hence, for an individual, the linear predictor in the hazard changes at the time of each of their repeat measurements when their random effects are updated using a new mixed model. Their random effects are estimated using only data up to that repeat measurement for individuals still under follow‐up at that repeat.

C‐indices and differences in C‐indices were calculated using the somersd package in Stata 25.

### 4.2 Estimation and validation

**Figure 1:**
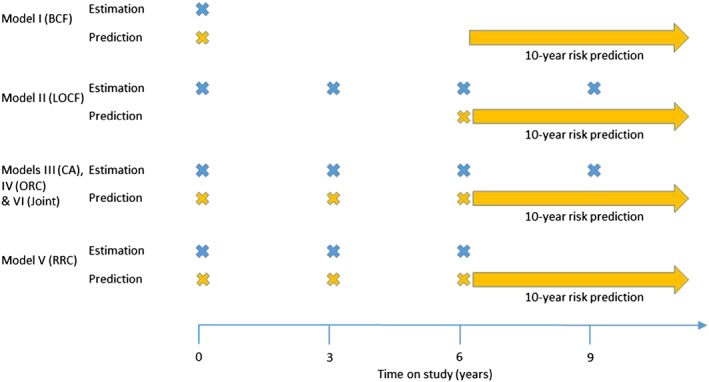
Data usage for models I–VI in estimation and prediction when making a 10‐year risk prediction from 6 years in the ARIC study (1996–1998). BCF, baseline carried forward; LOCF, last observation carried forward; CA, cumulative average; ORC, ordinary regression calibration; RRC, risk‐set regression calibration; Joint, joint model.

If, in the dataset used to estimate the model, a prediction is made for an individual before their event/censoring time, then the ORC and joint models could potentially use future covariate information from **X**_*i*_ in order to derive the prediction. In addition, the joint model will also use the future event/censoring time in order to obtain the random effects and predicted biomarker values. Therefore, predictions are likely to be over‐optimistic. To avoid this issue, we also fit the six models to datasets in which random 5000 individuals are censored for both their longitudinal and survival follow‐up just after the prediction times of interest. Only this ‘validation’ sample is then used to assess the predictive accuracy of the dynamic risk predictions by calculating risk predictions beyond their censoring date. All measures of predictive accuracy were therefore calculated amongst the 5000 individuals in the ‘validation’ sample using their actual (but unknown to the model) survival times. To illustrate, Figure 1 shows a schematic of the data that are used in the estimation and prediction for a 10‐year risk prediction being made after 6 years on study (i.e. 1993‐1995 in ARIC). The estimation data shown relate to the estimation of the longitudinal and survival model parameters relevant for a risk prediction at 6 years. We consider a BCF model where both the estimation and prediction sample use only the baseline measurement, allowing a comparison with the LOCF model where a repeat measurement of the biomarker is used rather than relying on a historical value. In practice, however, although a BCF model may be used for estimation, risk prediction would usually be undertaken using a contemporary measure of SBP.

### 4.3 Results

Log‐hazard ratios, C‐indices and Brier scores are shown in Table 1 for *L* = 3‐year and *L* = 10‐year survival prediction from *t*_*p*_ = 9 years on study. C‐indices and Brier scores have been estimated using individuals from the validation sample only. Log‐hazard ratio estimates for SBP from the BCF and LOCF models are lower than the CA log‐hazard ratio estimate; suggesting regression dilution bias due to measurement error has been partially corrected by the CA model. SBP log‐hazard ratios are even higher for the ORC, RRC and joint models that fully correct for measurement error. These log‐hazard ratios, however, are not directly comparable with those of the former models because they have been adjusted for SBP slope. Log‐hazard ratio estimates for the SBP slope were similar for the RRC and joint models, but lower for the ORC model. Bias in the ORC estimate may have been caused by event‐dependent drop‐out.

**Table 1:** Results from the Atherosclerosis Risk in Communities study: log‐hazard ratios for estimated current SBP (models BCF, LOCF and CA) or SBP intercept and SBP slope (models ORC, RRC and Joint), and C‐indices and Brier scores for 3‐year and 10‐year survival from 9 years on study (standard errors in brackets).

		Model					
		BCF	LOCF	CA	ORC	RRC	Joint
Survival model
logHR	SBP	0.019 (0.001)	0.019 (0.001)	0.022 (0.001)	0.028 (0.002)	0.027 (0.002)	0.030 (0.002)
	SBP slope	NA	NA	NA	0.076 (0.040)	0.139 (0.041)	0.155 (0.048)
3‐year CVD risk prediction
C‐index		0.750 (0.021)	0.742 (0.022)	0.751 (0.022)	0.752 (0.022)	0.750 (0.022)	0.751 (0.022)
Change in C‐index		Reference	− 0.009 (0.009)	0.000 (0.006)	0.002 (0.006)	0.000 (0.007)	0.001 (0.007)
Brier score		0.026 (0.002)	0.026 (0.002)	0.026 (0.002)	0.026 (0.002)	0.026 (0.002)	0.026 (0.002)
10‐year CVD risk prediction
C‐index		0.728 (0.012)	0.723 (0.012)	0.732 (0.012)	0.733 (0.012)	0.732 (0.012)	0.733 (0.012)
Change in C‐index		Reference	− 0.005 (0.005)	0.004 (0.003)	0.006 (0.003)	0.004 (0.004)	0.005 (0.004)
Brier score		0.100 (0.004)	0.100 (0.004)	0.099 (0.004)	0.099 (0.004)	0.099 (0.004)	0.099 (0.004)

Changes in C‐indices are compared with the BCF model. SBP, systolic blood pressure; RRC, risk‐set regression calibration; BCF, baseline carried forward; LOCF, last observation carried forward; CA, cumulative average; ORC, ordinary regression calibration; CVD, cardiovascular disease.

**Figure 2:**
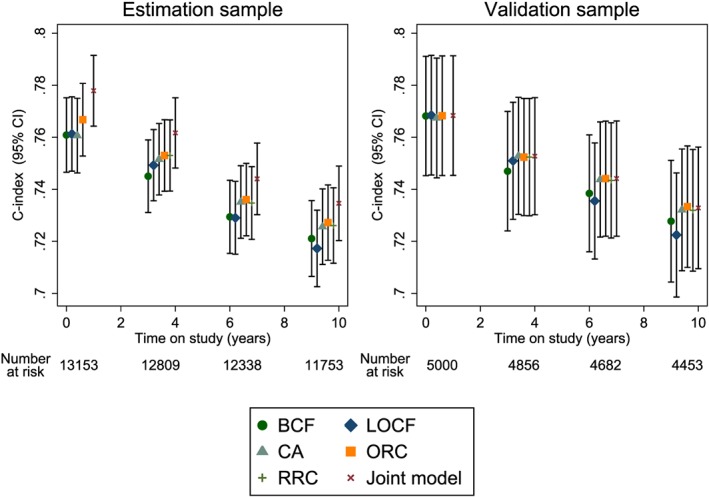
Dynamic C‐indices for the Atherosclerosis Risk in Communities study based on 10‐year risk predictions at different follow‐up times. BCF, baseline carried forward; LOCF, last observation carried forward; CA, cumulative average; ORC, ordinary regression calibration; RRC, risk‐set regression calibration; Joint, joint model.

Figure 2 shows C‐indices for 10‐year survival from *t*_*p*_ = 0, 3, 6 and 9years on study for both the estimation and validation samples. The C‐indices decrease over time, possibly because there tends to be less variability in baseline risk factors at later times as higher risk patients experience cardiovascular events early on. Over‐optimism in the C‐index using the estimation sample is particularly evident for the joint model, which incorporates survival information as well as future SBP measurements in the estimation of the random effects. C‐indices for all models were similar at baseline, but by year 9, the BCF and LOCF models have lower estimated C‐indices than the other four models. Table 1 also shows the C‐indices and changes in C‐indices for all models compared with the BCF model at year 9 for 3‐year and 10‐year risk predictions. The CA, ORC, RRC and joint models, which use information from repeat measurements, gave only slight improvements in the 10‐year C‐index compared with the BCF model. The CA model performed equally well compared with the more complex two‐stage and joint models. Results for the 3‐year C‐indices suggested all models performed equally well, but standard errors were large as fewer events were observed over the 3‐year prediction period than the 10‐year prediction period (116 events over 3years compared with 411 events over 10years).

**Figure 3:**
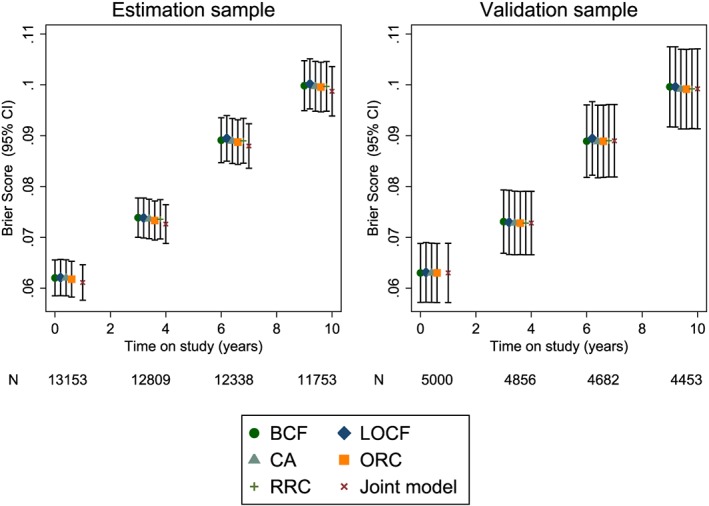
Dynamic Brier scores for the Atherosclerosis Risk in Communities study based on 10‐year risk predictions at different follow‐up times. BCF, baseline carried forward; LOCF, last observation carried forward; CA, cumulative average; ORC, ordinary regression calibration; RRC, risk‐set regression calibration; Joint, joint model.

Figure 3 shows dynamic Brier scores for the estimation and validation samples for the 10‐year risk predictions. Again, there is some over‐optimism in the joint model results using the estimation sample. For the validation sample, Brier scores are similar for all models. Dynamic calibration plots for the validation sample were also constructed and again showed very little difference between the models (results not shown).

## 5 Simulation study

To corroborate the findings found in the ARIC study, we conducted a simulation study. Longitudinal and survival data were simulated for 5000 individuals following a joint model with a random‐intercept and random‐slope that were independently associated with the hazard of the event:

$$\begin{array}{cc} x_{ij} & =(\beta_{0}+b_{0i})+(\beta_{1}+b_{1i})t_{ij}+\varepsilon_{ij}\\ {\left(b_{0i},b_{1i}\right)}^{T} & ∼N_{2}(0,\Sigma ),\;\varepsilon_{ij}∼N\left(0,\sigma_{\varepsilon }^{2}\right),\\ \Sigma & =\left(\begin{array}{ll} \sigma_{0}^{2} & \rho \sigma_{0}\sigma_{1}\\ \rho \sigma_{0}\sigma_{1} & \sigma_{1}^{2} \end{array}\right),\\ h_{i}(t) & =\lambda \exp\left(\alpha_{0}b_{0i}+\alpha_{1}b_{1i}\right),\\ C_{i} & =15,\\ T_{i}^{*} & =\min(T_{i},C_{i}). \end{array}$$

Longitudinal measurements were taken at four time points; *t* = 0 (baseline), *t* = 3,*t* = 6 and *t* = 9 years for each individual, with any longitudinal measurement after the event time $T_{i}^{*}$ excluded. An administrative censoring time of *C*_*i*_ = 15 years was imposed for all individuals. Each of the models was fit to the simulated datasets, and in addition, a survival model that uses the ‘true’ random effects *b*_0*i*_ and *b*_1*i*_ was also fit to show the best attainable discrimination. The dynamic C‐index was then calculated at four prediction times corresponding to each of the four visit times (*t*_*p*_ = 0,3,6,9). All individuals still at risk at these prediction times were used, and the C‐index was calculated based on a 5‐year risk prediction from the prediction time, whereby individuals were censored at *t*_*p*_ + 5 if their observed survival time was greater than this time horizon.

Each simulated dataset was further manipulated to create an additional four validation datasets whereby a random 2000 out of 5000 individuals were censored for their failure event just after the prediction time of interest (for *t*_*p*_ = 0,3,6,9) if their event/censoring time had not already occurred. Any longitudinal measurements taken after the prediction time were also discarded. Each model was then re‐fitted to each of the validation datasets, and the C‐index was calculated for each model based on the predicted 5‐year risk for the individuals who were censored at the prediction time and compared with their known (but not used in the estimation) survival time.

This process was repeated for 200 simulations, and bias and coverage statistics were obtained for the estimated model parameters, and the average C‐indices were calculated. Empirical and model‐based standard errors were compared for all parameters.

### 5.1 Scenarios

A number of possible scenarios were investigated using different parameter values in the data‐generating model. These are shown in Table 2; changes between the scenarios are shown in bold. In scenario 1, the true parameter values were set to be similar to those estimated from the ARIC study using SBP as the longitudinal biomarker and CVD as the event. Specifically, a mean baseline SBP of 120 mmHg (between‐person SD = 15 mmHg) with an average increase in SBP of 0.3 mmHg/year (between‐person SD = 1.0 mmHg/year) was used. Within‐person variability was considered high (SD = 10 mmHg). An exponential survival model was assumed for the hazard of CVD with a baseline rate of failure of 5/1000 person‐years. The hazard ratio for a 1 SD increase in baseline SBP was chosen to be 1.69 (1.04/mmHg increase) and 1.16 per SD increase in rate of change of SBP (1.16/mmHg/year increase). In scenario 2, the between‐person variability in the rate of change in SBP was increased to 5 (i.e. half of the within‐person standard deviation). This had the effect of making rate of change a more important predictor in the survival model because the hazard ratio per SD increase in rate of change of SBP was increased in magnitude to 2.12. In scenario 3, the baseline hazard was increased substantially so that the cumulative incidence of an event was approximately 20% by 15 years. In comparison, scenarios 1 and 2 had a cumulative incidence of between 2–2.5% by 15 years. The rationale for this scenario is that a higher event rate should lead to more event‐dependent (informative) censoring; hence, this scenario should favour the joint model. Finally, in scenario 4, the rate of change in SBP was uncorrelated with the underlying baseline SBP level in order to assess whether models that incorporated both these independent risk factors (i.e. ORC, RRC and joint) offered better predictive ability.

**Table 2:** Parameter values used in each of the simulation scenarios.

	Scenario			
Parameter	1	2	3	4
*β*_0_	120	120	120	120
*β*_1_	0.3	0.3	0.3	0.3
*σ*_*e*_	10	10	10	10
*σ*_0_	15	15	15	15
*σ*_1_	1	**5**	**5**	**5**
*ρ*	− 0.2	− 0.2	− 0.2	**0**
*λ*	0.005	0.005	**0.06**	0.005
*α*_0_	0.035	0.035	0.035	0.035
*α*_1_	0.15	0.15	0.15	0.15

Changes between the scenarios are shown in bold.

### 5.2 Simulation results

Bias and coverage for the 95% confidence intervals are shown in Table 3. The log‐hazard ratio for the biomarker at baseline, *α*_0_, is underestimated using the naive BCF and LOCF models and to a lesser extent using the CA model. Possible causes of this bias are regression dilution, because both BCF and LOCF use the observed biomarker measurements as predictors, and confounding due to the slope effect. The CA model utilises repeat biomarker measures, and hence, the bias is reduced. The two‐stage regression calibration models and the joint model provide near unbiased estimates for *α*_0_ in scenarios 1 and 2; however, both regression calibration models produce severely biased estimates of the hazard ratio for the biomarker rate of change. Coverage rates are generally poor for all models except the joint model. The low coverages are a factor of the large sample size, and thus small standard errors from each of the models.

**Table 3:** Bias and coverage results for log‐hazard ratios.

			Model					
Parameter	Scenario	True REs	BCF	LOCF	CA	ORC	RRC	Joint
			Bias					
*α*_0_	1	0.000	− 0.012	− 0.012	− 0.006	− 0.002	− 0.003	0.001
	2	0.000	− 0.018	− 0.016	− 0.003	− 0.002	− 0.003	0.000
	3	0.000	− 0.021	− 0.016	− 0.008	− 0.006	− 0.007	0.000
	4	0.000	− 0.012	− 0.014	− 0.001	0.001	− 0.000	0.000
*α*_1_	1	− 0.001	NA	NA	NA	− 0.145	− 0.120	0.020
	2	0.001	NA	NA	NA	− 0.044	− 0.041	0.001
	3	0.000	NA	NA	NA	− 0.063	− 0.068	0.000
	4	0.001	NA	NA	NA	− 0.047	− 0.044	0.001
			95% Coverage					
*α*_0_	1	92.0	1.5	1.5	45.0	90.5	84.5	92.5
	2	92.5	0.0	0.0	65.0	89.0	86.5	91.0
	3	94.5	0.0	0.0	0.0	3.0	0.0	95.5
	4	95.0	0.0	0.0	90.0	94.0	92.5	93.5
*α*_1_	1	95.5	NA	NA	NA	68.0	0.0	96.0
	2	95.0	NA	NA	NA	0.0	0.5	95.0
	3	94.5	NA	NA	NA	0.0	0.0	95.0
	4	94.0	NA	NA	NA	0.0	0.0	93.0

NA, not applicable; REs, random effects; RRC, risk‐set regression calibration; BCF, baseline carried forward; LOCF, last observation carried forward; CA, cumulative average; ORC, ordinary regression calibration.

**Figure 4:**
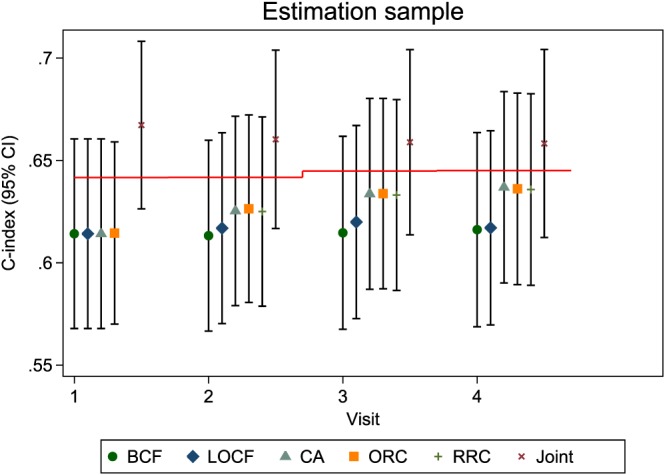
Dynamic C‐indices for simulation scenario 1 in the estimation sample. The red horizontal lines represent the mean C‐index under a model that uses the true random effects. BCF, baseline carried forward; LOCF, last observation carried forward; CA, cumulative average; ORC, ordinary regression calibration; RRC, risk‐set regression calibration; Joint, joint model.

The estimated dynamic C‐indices for each of the models applied to the estimation sample are shown in Figure 4, for scenario 1. The performance of the joint model is over‐optimistic when evaluated on data used in the model estimation process, because future survival events affect the estimated random effects through the shared parameter structure. Indeed, the predictive discrimination is even better than a model that uses the true random effects (shown by a red line in Figure 4).

**Figure 5:**
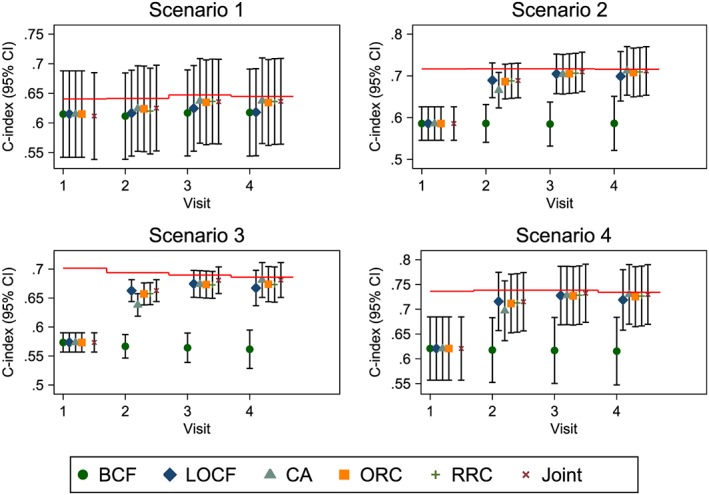
Dynamic C‐indices for simulation in scenarios 1–4. The red horizontal lines represent the mean C‐index under a model that uses the true random effects. BCF, baseline carried forward; LOCF, last observation carried forward; CA, cumulative average; ORC, ordinary regression calibration; RRC, risk‐set regression calibration; Joint, joint model.

Figure 5 shows the C‐indices for each of the four scenarios when considering just the validation sample. In scenario 1, there is a slight improvement in the C‐index for the CA, ORC, RRC and joint models over the naive BCF and LOCF models when using two or more repeat measures, suggesting that using the repeat measures to account for regression dilution can improve predictive accuracy. However, there is little to choose between the more complex models, with the simple CA model performing similarly to the more complex joint model.

In scenario 2 whereby the rate of change in the biomarker has a larger effect on the hazard, the baseline‐only model performs poorly at each evaluated prediction time. There is a slight improvement in the C‐index as model complexity increases and a large improvement as the number of repeat measures increases, although the biggest gain is seen when going from one biomarker measurement to two.

A similar conclusion is drawn in scenario 3 when the event rate is increased, with the CA and joint models performing slightly better than the regression calibration and LOCF models, and far better than the BCF model. In scenario 4, despite independence between baseline value of the biomarker and its rate of change, the dynamic updating of the hazard in the LOCF and CA models still captures enough information regarding baseline level/rate of change to give comparable predictive discrimination to the more complex models.

The mean Brier scores from each model in each of the four scenarios were also obtained, and results were similar between the six models, except in scenario 3 where the CA and joint models had slightly lower Brier scores (results not shown).

## 6 Discussion

We have investigated various models that incorporate data from repeat measurements of SBP into cardiovascular risk prediction. The models vary in complexity from the commonly used BCF and LOCF models to the joint model. Models differ in the extent to which they correct for measurement error and account for informative observation because of cardiovascular events, while the joint model also allows the propagation of uncertainty in the estimation of random effects. By including the BCF model in this paper and contrasting to the LOCF model, we highlight any improvement that is made in risk prediction by taking a repeat measurement of the biomarker rather than relying on a historical value. In the simulation study, where data were generated from a joint model, we found that, as expected, the joint model was unbiased but all other models gave biased hazard ratio estimates for the level and/or slope of the biomarker. In the ARIC study, we observed modest improvements in discrimination and calibration for models utilising information from repeat measurements, but little further improvement between the simpler CA model and the more complex two‐stage and joint models.

Our results therefore suggest that the more complex models are not delivering improvements in CVD risk prediction, even for simulated scenarios with more variability in individual slopes. Because most standard CVD risk factors (e.g. total and HDL‐cholesterol, lipid ratios) are relatively stable over time, at least in adulthood, our findings indicate that simple models that account for measurement error of these markers may be sufficient. Longer term follow‐up of SBP from a younger cohort would enable us to identify the importance of life‐course SBP trends in CVD risk prediction.

There are a number of possible caveats to these findings. Firstly, we have concentrated on the assessment of predictive discrimination using the C‐index, a measure that is known to be insensitive to detecting small differences in discriminative ability between two models 18. However, our sample size is large, and we also investigated predictive accuracy using Brier scores, which gave qualitatively similar conclusions. We used Harrell's estimator of the C‐index throughout 18, which may be biased in the presence of censoring 26, although this is unlikely to be a major issue for model comparisons that are all made using the same data. The true C‐index can be calculated if the variance of the linear predictor is known (see Appendix C of 27). As a check, we used this result and verified from the simulations for the BCF model that the true C‐index was indeed similar to the mean of Harrell's C‐index presented in the results. Secondly, our analyses could be underpowered to detect improvements from models that utilise repeat measures, as only four repeat measurements were observed. This, however, generally resembles current clinical practice for CVD risk prediction although future use of electronic health records may provide more rich historical data. Although others have shown clear associations with SBP trajectories in relation to the burden of subclinical atherosclerosis 28 and lifetime risk of CVD 29, variability in SBP measurements is likely to be dominated by day‐to‐day rather than long‐term variation, and hence, such a marker may not fully expose the utility of the longitudinal models being assessed here. It is therefore possible that the model comparisons could differ in other disease areas where there is a highly predictive biomarker that varies considerably over time, for example, prostate‐specific antigen for prostate cancer 30. The performance of the simple prediction models may also worsen if the predictive biomarkers have complex or nonlinear trajectories; this may be evident in a study if, say, the prescription of blood pressure lowering medication increased during follow‐up. Another limitation of our work is that deaths from other causes were assumed to be unrelated to CVD events 31. A joint model that accounts for the competing risk of non‐CVD deaths could be used 32 but is beyond the scope of this paper.

For the two‐stage and joint models, we have used random‐intercepts and slopes as time‐independent predictors in the survival models. An alternative, and perhaps more natural, parameterisation of the random effects would be to use an individual's current value of SBP and the SBP slope. But obtaining survival predictions from such a parameterisation is difficult because the current value of SBP is a function of time, and numerical integration of each individual's hazard is therefore required to calculate their survival function. The most appropriate parameterisation to use for prediction remains an open question. For example, Sène *et al.* found that a model with random effects as predictors gave better predictive accuracy than models with current value parameterisations in an application to prostate cancer 33.

The ORC and joint models both use future information to estimate the random effects, which then are used as predictors in the survival model, whereas the RRC model excludes future data by construction. For models that use future data, over‐optimism in the predictive accuracy can be avoided by using separate datasets for model estimation and validation. Cross‐validation could be used in cases where there is limited data available. It is likely that future trajectories are relevant in determining whether an event will take place. For models incorporating nonlinear effects of time, therefore, it may be that the use of future data in model estimation could aid event prediction for new individuals.

In our estimation sample, we have fitted a single survival model from baseline using either predictors that are updated with each measurement or using time‐independent predictors that are estimated based on all the longitudinal data. An alternative approach used for survival predictions, which explicitly separates the past from the future, is landmarking 9. A basic landmarking approach fits a series of survival models, one from each prediction time, with predictors that are estimated using only past measurements. Survival follow‐up is censored at the end of the prediction window. A super‐landmarking approach fits a single stratified survival model by stacking the data over all the prediction times. An individual event may therefore contribute multiple times if the individual is at risk at multiple prediction times. An important distinction between the two approaches is that, for our approach, predictors may be treated as time‐dependent variables in the survival model, while for super‐landmarking, predictors must be assumed to be constant beyond the landmark age. A landmarking approach could be used in conjunction with the LOCF, CA or ORC models described here but is distinct from joint models, which require simultaneous analysis of the repeat measurements and survival data. We have only considered survival models where the at‐risk period starts from baseline to enable comparisons with a joint model.

We leave for future work the investigation of models incorporating multiple time‐varying biomarkers. In cardiovascular risk prediction, for example, using repeat measurements of total and HDL‐cholesterol as well as SBP may improve prediction. Modelling the correlation between different biomarkers can add to the complexity of such models. The most popular joint modelling packages do not currently allow for the inclusion of even multiple uncorrelated longitudinal processes.
